# *P*henobarbital as an *A*djuvant to benzodiazepines when compared to *S*ingle-agent benzodiazepine *T*reatment (usual care) for *A*lcohol withdrawal syndrome in the intensive care unit (PASTA): a study protocol

**DOI:** 10.1186/s40814-026-01867-x

**Published:** 2026-06-11

**Authors:** Haustine Patt Panganiban, Samuel G. Ricciardone, Tess Evans, Melissa J. Ankravs, Timothy N. Fazio, Nicolas Clark, Aneil Avasthi, Christopher M. MacIsaac, Jeffrey J. Presneill, Emily J. See, Matthew W. Semler, Theodore J. Iwashyna, Adam M. Deane

**Affiliations:** 1 Department of Critical Care, Melbourne Medical School, The University of Melbourne, Melbourne, Australia; 2 Department of Intensive Care, The Royal Melbourne Hospital, Melbourne, Australia; 3 Centre for Clinical Research, Faculty of Health, Medicine and Behavioural Sciences, The University of Queensland, St Lucia, Australia; 4 Department of Pharmacy, The Royal Melbourne Hospital, Melbourne, Australia; 5 Electronic Medical Records (EMR) Team, The Royal Melbourne Hospital, Melbourne, Australia; 6 Clinical Informatics Centre, The Royal Melbourne Hospital, Melbourne, Australia; 7 Department of Addiction Medicine, The Royal Melbourne Hospital, Melbourne, Australia; 8 Disease Elimination Program, Burnet Institute, Melbourne, Australia; 9 Australian and New Zealand Intensive Care Research Centre (ANZIC-RC), School of Public Health and Preventive Medicine, Monash University, Melbourne, Australia; 10 Department of Intensive Care, Austin Hospital, Melbourne, Australia; 11 Vanderbilt Institute of Clinical and Translational Research, Vanderbilt University Medical Centre, Nashville, USA; 12 Division of Pulmonary, Allergy, And Critical Care Medicine, Department of Medicine, Vanderbilt University Medical Centre, Nashville, USA; 13 Department of Pulmonary & Critical Care Medicine, Johns Hopkins University, Baltimore, USA; 14 Department of Health Management and Policy, Johns Hopkins Bloomberg School of Public Health, Baltimore, USA

**Keywords:** Alcohol withdrawal syndrome, Benzodiazepines, Clinical Decision Support Systems, Critical illness, Electronic medical records, Phenobarbital, Randomized controlled trial, Embedded research

## Abstract

**Background:**

Abrupt cessation of alcohol consumption following hospital admission can induce alcohol withdrawal syndrome. This syndrome is challenging to manage and is associated with considerable morbidity and mortality. Current management of alcohol withdrawal syndrome in Australia is generally titrated benzodiazepine administration. In some regions, phenobarbital is preferred but evidence to support its superiority is weak.

**Methods:**

We will conduct a single-center, three-arm, open-label, parallel-group, randomized clinical trial. Our primary objective is to determine the feasibility of embedding a randomized clinical trial within an electronic medical record to compare phenobarbital to single-agent benzodiazepine for patients with alcohol withdrawal. We will screen and randomize 45 patients using the Epic electronic medical record system. Patients will receive either low- or standard-dose intravenous phenobarbital (4 or 8 mg per kilogram), or usual care (benzodiazepine regimen). The primary outcomes to assess feasibility are usability, screening rates, enrollment rates, and compliance. Secondary outcomes are exploratory and include alcohol withdrawal scale scores, dose of benzodiazepine administered, health utilization, adjuvant drug treatments, and patient outcomes.

**Discussion:**

There are limited high quality data evaluating the use of phenobarbital administration for alcohol withdrawal syndrome. Additionally, there is limited data evaluating the embedding of screening, randomization, and administration of a sedative drug within the electronic medical record. Our feasibility trial will establish whether this is possible within our health care system. In this protocol paper we detail how we will embed this trial within the electronic medical record Epic.

**Trial registration:**

The study (RMH2024.320 v2 06/01/2025) is registered with Australian New Zealand Clinical Trials Registry (ANZCTR) ACTRN12625000320459. Registered on 17 April 2025. Trial Sponsor: The Royal Melbourne Hospital.

**Supplementary Information:**

The online version contains supplementary material available at 10.1186/s40814-026-01867-x.

## Background

Alcohol is the most used and misused drug globally [1]. In Australia, approximately 30% of the population aged greater than 14 years of age use alcohol in a way that puts their health at risk, with more than 80,000 alcohol-related hospitalizations annually [2].

Hospital admission results in immediate cessation of alcohol consumption for individuals with an alcohol use disorder, which risks precipitating the alcohol withdrawal syndrome [3]. Alcohol withdrawal syndrome causes symptoms that are distressing for patients, their families and health care workers. The symptoms include tremors, irritability, insomnia, diaphoresis, tachycardia, and fever [4]. Twenty percent of those with alcohol withdrawal syndrome develop severe symptoms. Features of severe alcohol withdrawal include delirium tremens and withdrawal seizures [4]; severe alcohol withdrawal markedly increases the risk of death [3]. It is, therefore, critical that severe alcohol withdrawal syndrome is prevented and/or treated urgently [3].

### Treatment of alcohol withdrawal

In Australia, benzodiazepines remain the default drug class for the prevention and treatment of alcohol withdrawal syndrome [5]. Benzodiazepines reduce withdrawal severity, incidence of delirium and frequency of seizures [5]. However, in some cases of severe alcohol withdrawal syndrome, even extremely large doses of the benzodiazepine diazepam (e.g., ≥ 120 mg/day) may not achieve adequate clinical response [6]. Patients with severe alcohol withdrawal syndrome can also exhibit tolerance to benzodiazepines, and cumulative doses of benzodiazepines risk respiratory depression, falls, and extended delirium [7–9].

Phenobarbital is a barbiturate drug that is inexpensive and has a long half-life. The clinical introduction of barbiturates began in 1904 and caused great change in the management of psychiatric and neurological disorders at the time [10]. Some studies have proposed that phenobarbital may be superior to benzodiazepines for the treatment of alcohol withdrawal syndrome [11–14]. There is variation in the treatment of alcohol withdrawal but in some regions, phenobarbital is the preferred drug; however, there is no high-quality evidence that phenobarbital is superior to a benzodiazepine regimen [11–13, 15]. Umar and colleagues conducted a systematic review and meta-analysis of all studies that evaluated the use of phenobarbital when compared to other strategies in patients admitted to an intensive care unit (ICU) for alcohol withdrawal syndrome [11]. Of nine studies identified by their systematic review, one was a retrospective case series (11 participants) and eight were retrospective observational studies (31 to 135 participants). The pooled mean differences in duration of hospital admission with phenobarbital compared to control were − 2.6 days (95% CI, − 4.9 to − 0.7). Regarding duration of ICU admission, the pooled mean difference was − 1.17 days (95% CI, −1.17, 0.09, *n* = 6 studies, *n* = 624 participants) in patients who received phenobarbital compared to control. While the difference in risk of intubation was not statistically significant between groups (relative risk, 0.52 (95% CI, 0.25 to 1.08), *n* = 6 studies, *n* = 624 participants), the point estimate suggested that intubation was less common with phenobarbital.

A recently published study described the implementation of a phenobarbital monotherapy order set for alcohol withdrawal syndrome in a single-center setting [16]. A total of 254 patients were included in the retrospective analysis, including 154 before and 100 after the phenobarbital order set was implemented. Prior to order set implementation, 33.8% of patients received a median (IQR) of 3.7 (1.9–10.5) mg/kg of phenobarbital cumulative, following implementation, 71.0% received a median (IQR) 12.6 (9.7–16.1) mg/kg [16]. A significant improvement in clinical outcomes was reported following phenobarbital order set implementation. Alcohol withdrawal treatment duration and time to hospital discharge were both significantly reduced following order set implementation by 30.1 h (*p* < 0.001) and 2.2 days (*p* = 0.004), respectively [16]. Between groups, alcohol withdrawal scale scores were similar in the first 24 h but were significantly lower after order set implementation on subsequent days of treatment [16]. Safety outcomes, including prolonged mechanical restraints, new intubation, and in-hospital mortality did not differ between groups.

There is no randomized clinical trial conducted in an ICU to evaluate the effects of phenobarbital for alcohol withdrawal. An experienced group attempted to assess the feasibility of conducting an allocation-concealed, blinded, randomized controlled trial comparing symptom-triggered benzodiazepine therapy with either a single dose of adjuvant intravenous phenobarbital (7.5 mg per kilogram ideal body weight), or a single dose of matching intravenous placebo for patients with alcohol withdrawal syndrome [17]. This feasibility trial (Phenobarbital for Severe Acute Alcohol Withdrawal Syndrome (PHENOMANAL) NCT03586089) was terminated due to a difficulty in recruiting patients (personal communication). This may be because in North America the use of phenobarbital is widespread. However, in Australia, phenobarbital use is not yet established. Accordingly, it may be possible for us to systematically test the repurposing of this medication for alcohol withdrawal syndrome.

### Embedding critical care trials within electronic medical records trials

Electronic medical records offer the potential to significantly increase the efficiency of conducting clinical trials [18, 19]. Using electronic medical records can enhance screening, recruitment, treatment ordering, monitoring, and data collection, to improve feasibility, and reduce trial expense [20, 21]. Despite its potential, few electronic medical record-embedded clinical trials have been conducted in the ICU [22, 23].

Our group has embedded trial activities within the electronic medical record [20]. While we have utilized the electronic medical records for screening, randomization and encouraging compliance with allocated treatment, we have never used this approach to evaluate the effect of a sedative drug. This is relevant as it is conceivable that clinicians may be less compliant following electronic medical record guidance in recommending the use of a sedative drug, particularly one with which they are unfamiliar. Furthermore, in prior protocols we have used a simple randomization schedule within the electronic medical record. This trial will evaluate if more sophisticated approaches to randomization can be implemented.

There is a recent report describing the implementation of a phenobarbital order set within an electronic medical records system (Epic Systems Corporation) for all patients admitted to a community hospital with alcohol withdrawal [16]. This retrospective audit reported the phenobarbital order set was frequently used and when compared to historical controls reduced time to hospital discharge [16].

In summary, it is unknown whether we can conduct a randomized clinical trial to evaluate the use of a sedative drug, such as intravenous phenobarbital, to treat alcohol withdrawal and embed all research processes within an electronic medical record.

## Methods

### Study objectives

Our primary aim is to determine the feasibility of embedding this intervention within an electronic medical record platform. Feasibility will be assessed by usability, screening, randomization, and compliance with treatment allocation. The criteria and thresholds are described in greater detail below (see “Outcome measures”).

Our secondary aims are to, between groups, compare specific outcomes for:

1. The effect on alcohol withdrawal scale scores over time.
2. The dose of diazepam administered.
3. The effect on health utilization.
4. The effect on patient outcomes—(delirium/coma-free days), seizures, respiratory depression, need for tracheal intubation, and death.

These outcomes are exploratory and will be reported as point estimates and 95% confidence intervals.

### Screening

Screening will be performed automatically within the electronic medical record using a Clinical Decision Support System called “*Our Practice Advisories*”. The *Our Practice Advisories* were built to include local rules and logic that contain the study inclusion criteria. Once a patient meets all inclusion criteria the *Our Practice Advisory* will trigger but should not interruptive the physician’s workflow. The screened patient will be counted using a real-time electronic medical records reporting called “*Reporting Workbench*” and a real-time messaging tool called “*In-Basket*”.

### Study participants

Forty-five participants will be included in this trial. All patients admitted to the Intensive Care Unit will be screened using the eligibility criteria detailed in Table 1.

**Table 1:** Eligibility criteria

**Inclusion criteria**
≥ 18 years old
Admitted to ICU
Treatment ordered for alcohol withdrawal syndrome, defined as an order for > 12 mg of diazepam (or equipotent benzodiazepine) within a 6-h period or > 40 mg of diazepam within a 24-h period
Witnessed seizure(s) deemed by clinicians to be suspected or in known relation to alcohol misuse, regardless of previous benzodiazepine administration
**Exclusion criteria**
> 75 years old
Pregnant or breast feeding
Documented liver cirrhosis or severe liver disease (indicated by an INR > 5.0)
A cause other than alcohol withdrawal is thought to be more likely for delirium
Admission with polypharmacy overdose and substantial co-ingestion of a CNS depressant drugs (opioids, benzodiazepines, quetiapine, toxic alcohol ingestion, or gamma-hydroxy-butyrate) is documented
Prescribed phenobarbital prior to admission
Known allergy or hypersensitivity syndrome to phenobarbital
Clinician deems is highly likely to discharge against medical advice or die in the next 24 h
Treatment limitations precluding endotracheal intubation
Acute kidney injury (stage 3)
Current treatment with drug known to interact with phenobarbital, i.e., ticagrelor, prasugrel, warfarin, enteral/parenteral calcineurin inhibitor, or HIV-protease inhibitor
History of porphyria
History of myasthenia gravis
Allergy or rash with other antiepileptics
Previous participation in this trial
Phenobarbital contraindicated for patient due to local product information
Inability to obtain intravenous access

### Eligibility assessment

Once a patient meets the eligibility criteria, the *Our Practice Advisory* alert will notify the physician of the study, and the eligibility criteria will be displayed for review. The physician will confirm patient eligibility using the steps outlined in Supplemental Material 1.

### Consent and ethical approval

Given all patients included in this study will have been admitted to the ICU with underlying diagnosis and treatment and have alcohol withdrawal syndrome and have received at least 12 mg of diazepam, they will lack capacity to provide informed consent prior to participation. Treatment of alcohol withdrawal is an emergency and is already provided to non-consenting patients when it is deemed to be in the patient’s best interest. Accordingly, it is not possible to identify and obtain consent from the medical treatment decision maker. Trial participants will continue to receive usual care or usual care and phenobarbital—which is the preferred drug at many international centers. Participants in this trial will be included based on Sect. 53 of the Medical Treatment Planning and Decisions Act (Victoria)—“Medical treatment and medical research procedures in an emergency”. The administration of intravenous phenobarbital in the setting of acute alcohol withdrawal syndrome is necessary, as a matter of urgency to prevent serious damage to the person’s health, and limit suffering, pain or distress. A waiver of consent is consistent with the Australian National Health and Medical Research Council (NHMRC) Statement on Ethical conduct in Human Research (chapter 2.3) [24].

The protocol and consent process were approved by the Royal Melbourne Hospital Human Research Ethics Committee (HREC114383). The protocol is registered with Australian New Zealand Clinical Trials Registry (ACTRN12625000320459).

### Enrollment, randomization, and allocation concealment

Once the physician confirms eligibility, enrollment occurs in the electronic medical records. This will be performed by creating an action extension record attached to the *Our Practice Advisory* that triggers silently within the system to prevent interruption (see Supplemental Material 1).

For this trial, we will link the REDCap stratified randomization module to Epic [25, 26]. Enrolled patients will be randomized to groups using a 1:1:1 ratio allocation. The *Our Practice Advisories* will alert the physician that a patient is enrolled and provide a REDCap link to randomize (see Supplemental Material 1). If the physician cancels the advisory, the patient remains suitable for screening and the alert will reappear until the participant is assessed for eligibility. An instructional video on how to perform the randomization will be available via hyperlink within the *Our Practice Advisories* to guide the physician. The study team will monitor the REDCap randomization dashboard regularly to ensure consistent participant arm allocation between REDCap and the electronic medical records. In the event of randomization errors in which the physician did not enter the allocated arm correctly in the electronic medical records, the participants will be included in the intention-to-treat analysis but excluded in the per-protocol analysis (see scenario examples in Supplemental Material 1). The randomization sequence was created using the random function in Excel by a researcher independent of this trial and uploaded into REDCap. This sequence will be concealed from all physicians and will be restricted by setting user privileges in the REDCap project. The detailed REDCap project configuration is available in the Supplemental Material 1.

### Compliance with treatment allocation

The physician will transcribe the treatment arm allocation from REDCap to the electronic medical record alert (see Supplemental Material 1). Once the treatment arm option is selected, the system will assign a “*Smart Data Element*” that stores a number that signifies arm allocation at the patient record level. This system element is not visible to end-users and will only be visible to certified technical users with restricted access. Physicians will also be alerted when they are prescribing a drug included in the drug group being investigated and includes a study “*Order Set*” (see Supplemental Material 1). Compliance within the treatment allocation will be achieved by incorporating a well-defined system of rules and logic within the electronic medical records study *Order Set*. This will ensure that only the allocated treatment for a specific participant will be presented to the physician when ordering. The physician will have the capacity to override the alert and provide an override reason within the alert. The study team will monitor override reasons through a message sent to the “*InBasket*” (see Supplemental Material 1).

Randomized participants will be automatically enrolled within the electronic medical records into a study record and will send a real-time notification message via *InBasket* to the study team (see Supplemental Material 1). This study record will be used to monitor participant progress within the study. This will also be accompanied by a series of cross-arm *Our Practice Advisories* to prevent treatment deviation (see Supplemental Material 1).

### Design and intervention

This feasibility study protocol is reported according to the SPIRIT 2025 statement [27].

**Fig. 1:**
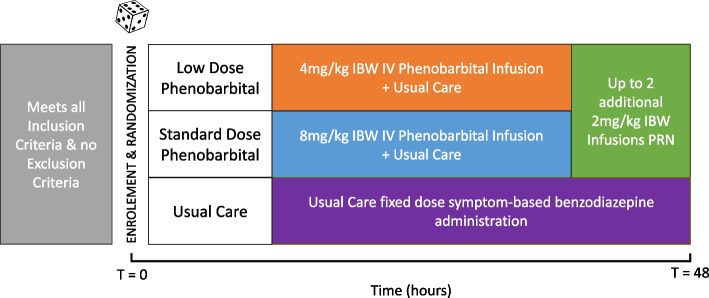
Simplified overview of study design

We will conduct a single-center, three-arm, open-label, parallel-group, randomized controlled trial. This trial will take place in a mixed medical-surgical-trauma ICU in Australia [28]. Eligible participants will be randomized to one of three arms; the overall study design is depicted in Fig. 1. The schedule of assessments is shown in the recommended SPIRIT Table (Table 2).

**Table 2:** Standard Protocol Items: Recommendations for Interventional Trials (SPIRIT) table summary

	**Trial period**				
	**Enrollment**		**Post-randomization**		**Close-out**
**Timepoint**	***Pre-eligibility***	***Eligibility***	***Up to 48-h post-enrollment***	** > *48-h post-enrollment***	***Data analysis***
**Enrollment:**					
**Eligibility screen**	X				
**Eligibility assessment**	X				
**Randomization**		X			
**Interventions**					
***Low/standard-dose phenobarbital administration***			X		
***Usual care***		X	X	X	
**Assessments:**					
**Demographic information**	X				
**Feasibility (primary outcomes—useability, screening rates, enrollment rate and compliance)**		X	X	X	X
**Secondary outcomes**		X	X	X	X
**Adverse events**		X	X	X	X

1. Low-dose phenobarbital (4 mg per kilogram ideal body weight), infused over 30 min in addition to usual care;
2. Standard-dose phenobarbital (8 mg per kilogram ideal body weight) over 30 min in addition to usual care; or
3. Usual care.

If allocated to phenobarbital arms, patients may also receive additional lower doses of the study drug once 30 min have elapsed from the initial load. Up to a maximum of two additional doses (2 mg per kilogram ideal body weight) may be given in a 48-h period after randomization.

### Rationale for dosing

For this trial we will use intravenous phenobarbital. Studies conducted outside of a critical care environment have used enteral or intramuscular dosing [29, 30]. However, intravenous administration of drugs is generally preferred in the emergency and critical care settings due to more predictable pharmacokinetics and onset of action.

Existing literature describes using intravenous phenobarbital for the treatment of alcohol withdrawal syndrome in either a weight-based or a fixed dose regimen. A retrospective cohort study included 179 adult patients admitted to a tertiary academic hospital with alcohol withdrawal syndrome and reported no difference in phenobarbital-related adverse drug events between 6 mg per kg and 10 mg per kg ideal body weight [31]. Of these 179 patients, the majority (~ 56%) received their initial phenobarbital load in the emergency department, a third (~32%) received initial phenobarbital in the ICU, and the remainder were treated in other inpatient wards [31]. A prospective blinded randomized trial of patients presenting to the emergency department with alcohol withdrawal syndrome compared intravenous phenobarbital to intravenous lorazepam [32]. Patients received 260 mg of intravenous phenobarbital initially. Subsequent doses of 130 mg were given at the discretion of the treating physician. Of the 25 patients who received phenobarbital. the mean number of doses was 2.9 (range 1 to 6) for a mean dose of 509 mg (range 260 to 910). A single centre retrospective study of 87 patients compared patients after a protocol change from a ‘low-intermittent’ phenobarbital dosing regimen to a ‘front loaded’ phenobarbital. During the initial period all patients with a high alcohol withdrawal syndrome score despite three intravenous doses of 8 mg of lorazepam (total) received a fixed dose of 260 mg followed by 130 mg intravenous push every 15 min as needed. After the protocol change, patients received a weight-based regimen 10 mg per kilogram actual body weight. The patients receiving the weight-based dosing (subsequent period) were significantly less likely to be mechanically ventilated and had a shorter duration of admission [33].

In our intervention arms we will test two weight-based dosing regimens. The doses chosen are slightly less than administered in the studies above. We have reduced doses because our clinicians are less familiar with using phenobarbital. We will also use ideal body weight rather than actual body weight [34]. In the lower- and standard-dose groups, patients will receive 4 and 8 mg per kg ideal body weight of phenobarbital, respectively.

### Usual care

At our institution, usual care for the management of alcohol withdrawal syndrome is guided by a hospital protocol. This policy recommends enteral administration of 20 mg of diazepam every 2 h when needed (i.e., PRN) based on withdrawal symptoms, with repeated dosing determined by alcohol withdrawal scale score. This policy reflects the standard for treatment for alcohol withdrawal Australia-wide, with the Australian Government classifying diazepam as a “gold standard” with an “A” strength of recommendation, and a “Ia” level of evidence but individual clinicians can modify prescriptions to lesser dose or the intravenous route [35]. Prescribing of diazepam can occur within the electronic medical records using an alcohol withdrawal syndrome order set. Oxazepam is considered for patients with relative contraindications to long-acting benzodiazepines, such as severe alcoholic hepatitis. Oxazepam 15 mg is approximately equipotent to 5 mg diazepam.

**Fig. 2:**
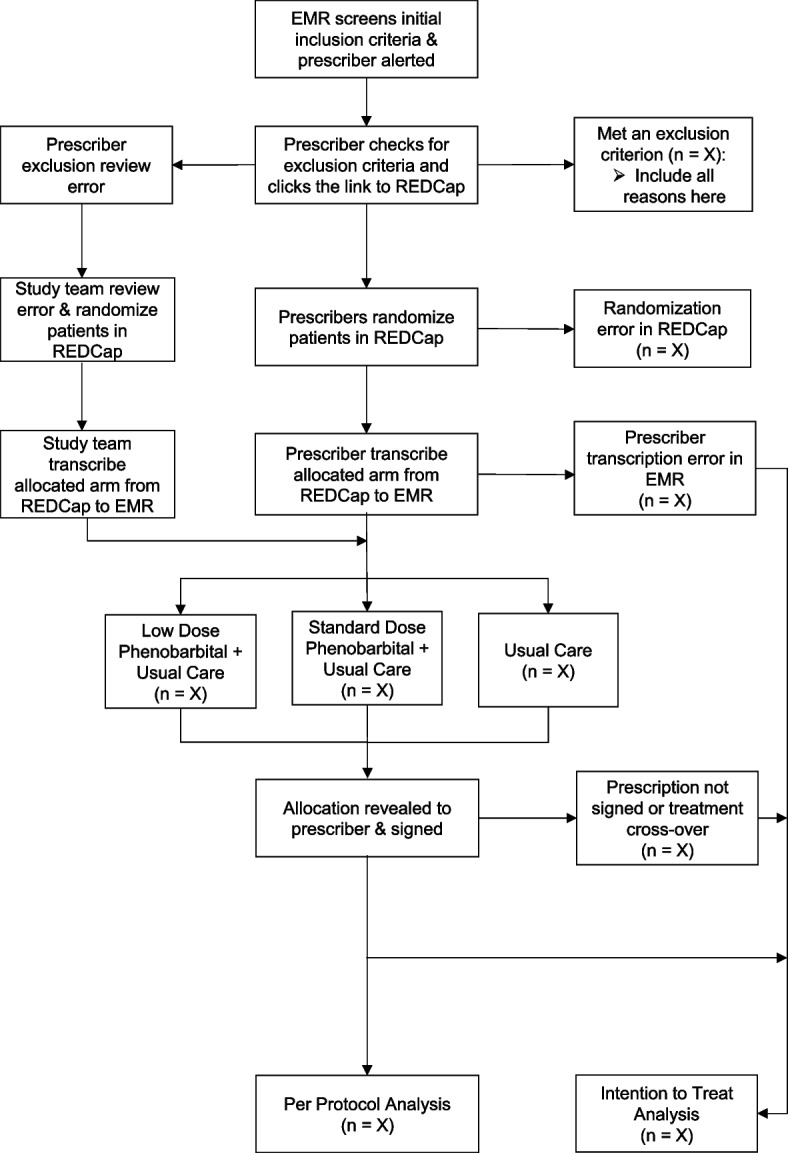
Patient flow diagram

Patient flow will occur as outlined in Fig. 2 below.

### Electronic medical records embedded trial design

The embedding process was designed with multiple key stakeholders from the ICU and electronic medical record team as detailed in Supplemental Material 1. We established proof-of-concept of embedding all aspects of a trial evaluating electrolyte administration using the Epic electronic medical records system [23]. Multiple iterations were performed to arrive at an acceptable design concept for this trial. Validation and co-design sessions were conducted before the implementation with pharmacists, physicians, and a physician informatician to collect feedback and incorporate it into the design. The final design was tested using multiple cycles following the electronic medical record standard test case scenarios. The design underwent governance and change control procedures to ensure system compliance. A training plan was delivered to ensure that the end-user is informed of the new functionality. During the implementation phase, the electronic medical record build records monitoring, enhancement, and maintenance will be conducted to ensure compliance with local hospital and precinct-wide system policy.

### Outcome measures

#### Primary outcomes

Feasibility will be determined by the feasibility outcomes: (1) Usability. (2) Screening rates. (3) Enrollment rate. (4) Compliance with treatment allocation.

Usability will be determined by conducting physician perceived electronic medical records usability survey using the System Usability Scale 10 item questionnaire (see Table 3) [36]. The System Usability Scale ranges from 0 to 100, with greater scores indicating better usability. Feasibility of screening will be determined as the electronic medical records identifying two patients per month who meet all eligibility criteria. Feasibility of enrollment and randomization will be defined as 70% of eligible patients being randomized (eligible patients determined by manual screening and in basket message alerts) and completion of the trial within 2.5 years. The thresholds for feasibility of compliance with treatment allocation is that 80% of patients allocated to phenobarbital receive it and < 10% of patients assigned usual care receive phenobarbital.

**Table 3:** System Usability Scale

	**Strongly disagree**	**Somewhat disagree**	**Neutral**	**Somewhat agree**	**Strongly agree**
1. I think I would like to use this alert frequently					
2. I found the alert unnecessarily complex					
3. I thought the alert was easy to use					
4. I think that I would need the support of a technical person to be able to comply with the alert					
5. I found the various functions in this alert were well integrated					
6. I thought there was too much inconsistency in this alert					
7. I would imagine that most people would learn to use this alert very quickly					
8. I found the alert very cumbersome to use					
9. I felt very confident acting on the alert					
10. I needed to learn a lot of things before I could get going with this alert					
**Additional questions**					
1. I found the instruction on randomization with the alert is easy to follow					
2. I feel very confident on randomizing the patient using the alert					
3. I think the instructional videos within the alert is helpful					
4. I feel comfortable in placing the study medication order using the alert					
5. I thought the whole randomization process is easy					

#### Secondary outcomes

- Cumulative alcohol withdrawal scale scores from study days 1–7. Alcohol withdrawal scale scores are reported in Epic by nurses and entered at least once per admission.
- Quantity of diazepam administered will be extracted from the medical records. Diazepam (or equivalent) dosing will be extracted from Epic and reported as days received, dose per day and total dose.
- Health utilization include number of code grey events (code grey events—these are a hospital-wide response due to aggression or threatened/actual violence, episodes of harm to staff due to aggression or violence, and violence, duration of intensive care admission, duration of hospital admission, days of antibiotic therapy, use of mechanical restraints, need for additional staff on discharge to ward to manage behaviour (so-called Nurse Special)), and adjuvant drug treatments (including, clonidine, dexmedetomidine, olanzapine, quetiapine, haloperidol, and droperidol) [37]. Code grey events will be reported as actual number and mean per patient for 7 days after randomization. Occupational aggression and violence will be extracted from the medical records. Duration of intensive care admission from randomization (reported as days free of ICU at day 7). Duration of hospital admission from randomization (reported as days free of ICU at day 28). Days of antibiotic therapy for 7 days after randomization reported as binary (Y/N) and days received. Code grey events will be reported as actual number and mean per patient for 7 days after randomization. Use of mechanical restraints for 7 days after randomization and reported as binary (Y/N) and days received. Use of Nurse Special on discharge to ward and reported as binary (Y/N), Other adjuvant drug treatments in ICU (clonidine, dexmedetomidine, olanzapine, quetiapine, haloperidol, droperidol) for 7 days after randomization will be reported as received (Y/N)
- The effect on patient outcomes: delirium/coma-free days, seizures, respiratory depression, need for tracheal intubation, mortality). Delirium and coma-free days will be recorded using the process used previously [38]. A patient will be regarded as delirium/coma-free for that calendar day if: They are alive and discharged from the ICU or They are alive in the ICU with (all of the following): (1) the Richmond Agitation-Sedation Scale (RASS) score being − 2 to 0 all day at all time points; (2) no Confusion Assessment Method for the ICU (CAM-ICU) positive outcome recorded at any time point; and (3) no documentation of agitation during the calendar day
- For unintubated patients, frequency of intubation post randomization and for 7 days after randomization will be reported as yes/no.
- Ventilator days will be reported as median (range)
- Time to extubation
- Frequency of seizures will be reported (for 7 days after randomization) as the number of patients having one seizure and the total number of seizures.
- Incident neuroimaging (CT or MRI) in ICU and for 7 days after randomization
- Respiratory depression, counted as hypoxia (SpO2 < 92% on room air when previously > 95% in the context of respiratory rate < 10) or new hypercapnia (PaCO2 > 50 mm Hg)
- Sedation scores will be recorded using the Richmond Agitation Sedation Score. RASS scores are reported in Epic by nurses and reported at least once per nursing shift.
- Highest PaCO2 for calendar day will be recorded when available
- Falls during hospitalization for first 7 days (documented using Riskman)

### Data collection

As an embedded trial, eligibility screening, enrollment, randomization, and patient demographics will be collected in real time during hospital admission using the Epic reporting tool. This will serve as an operational data set for monitoring participant accruals and progress.

A study database will be created using REDCap case report forms (see Supplemental Material 1) hosted at The Royal Melbourne Hospital [25, 26]. Data that requires narrative interpretation will be collected manually from the electronic medical records and entered into case report forms by the delegated study team members.

### Data verification

All data will be kept securely for at least 15 years and stored as per Royal Melbourne Hospital research policy. Access will be password-controlled and limited to members of the study team. An ICU Research Coordinator independent of this trial will perform source data verification. Source data verification will be conducted for 100% of the primary outcomes (feasibility outcomes) and 100% of serious adverse events.

### Trial oversight and monitoring

The Trial Safety Committee includes intensive care physicians Adam Deane and Tess Evans and senior ICU pharmacist Melissa Ankravs. The Data Safety Monitoring Committee includes intensive care physician Matthew Maiden and addiction medicine physician Nazila Jamshidi.

The Trial Safety Committee will meet monthly to review all adverse events, recruitment, and compliance with the interventions. The Trial Safety Committee may decide to stop the trial if it is clearly not feasible despite attempts to resolve issues. Every three months the Data Safety Monitoring Committee will be provided a summary of all serious adverse events, adverse events, recruitment rates and compliance with the interventions. The Data Safety Monitoring Committee will use their own discretion to recommend stopping the trial due to adverse/serious adverse events.

Patients with alcohol withdrawal syndrome who are admitted to the ICU are at high-risk for adverse events. However, the drugs used as part of usual care, benzodiazepines, and the intervention, phenobarbital, have known adverse effects associated with their use. It is recognized that the patient population in the ICU will experience aberrations in laboratory values, signs, and symptoms due to the severity of the underlying disease and the impact of standard treatments [39]. These will not necessarily constitute adverse events or serious adverse events unless they are considered related to study treatment, or in the principal investigator’s clinical judgment are not recognized events consistent with the participants underlying critical illness and/or chronic diseases and expected clinical course. In Australian ICU trials, the reporting of adverse events for medicinal products is restricted to events that are considered related to study treatment (possibly, probably, or definitely).

For the purposes of this open-label trial, serious adverse events will only be reported if they occur in participants receiving phenobarbital. Serious adverse events be defined as drug reaction with eosinophilia and systemic symptoms (DRESS), Stevens-Johnson syndrome, toxic epidermal necrolysis (TEN), anaphylaxis (angioedema), tracheal intubation, cardiac arrest or pre-arrest, and death post randomization and prior to ICU discharge. Any of these events that considered to be identified as probably or definitely related to phenobarbital will be reported to the data safety monitoring committee within 48 h. Because events such as tracheal intubation and cardiac arrest can occur in the usual care group (i.e., diazepam as single agent drug), these will be collated and reported with adverse events.

For the purposes of this trial an adverse event will be considered new hypotension (mean arterial pressure < 60 mmHg or an increase of > 50% of vasoactive drug infusion) during infusion of phenobarbital for 30 min after infusion ceased, or ‘mild’ skin reaction (rash, hives, itch). Any adverse event thought to be study treatment related will be evaluated by a trial physician. Adverse events will be collated and reported to the Trial Safety Committee and Data Safety Monitoring Committee at three monthly intervals.

### Withdrawals

In the circumstance that the treatment physician believes usual care is clearly preferable over the allocated phenobarbital regimen, the treatment physician can override the treatment allocation. This will be recorded as a protocol violation with data retained for intention to treat analysis.

In the circumstance that the medical treatment decision maker (MTDM) refuses retention of data, all patient data will be removed from the database and the patient will be considered removed from the intention to treat analysis, if this occurs the participant will not be replaced.

### Sample size

Our feasibility trial will include 45 participants. It is reasoned that a sample size of 45 participants will ensure assessment of our feasibility metrics (usability, screening and enrollment rates, and compliance) and that the multidisciplinary team members gain experience with administration of phenobarbital for alcohol withdrawal.

In this pilot trial, an important feasibility outcome is an estimation of the screening rate needed for successful enrollment in the definitive trial, which was specified as two patients per month who meet eligibility criteria. An estimate of this parameter was desired with a precision of 1 (i.e., exclude rates less than one per month with 95% confidence, using a 95% one-sided estimate). Feasibility of enrollment and randomization is defined as 70% of eligible patients being enrolled and completion of the trial within 2.5 years.

A rate lower than one eligible patient per month would make a future study infeasible, but a higher rate (leading to a faster recruiting trial) was not important to exclude in this pilot study.

To estimate this recruitment rate with the desired level of precision (5% error, all one sided), calculations showed that 12 participants would be needed to be enrolled (taking 6 months at two per month) [40].

Thus, the present study aiming for 15 patients in each of three treatment groups (equals 45 patients in total) will exceed the minimum sample size necessary to exclude an infeasibly low recruitment rate, while also allowing preliminary estimates of some between-group parameters of interest.

### Statistical analysis

Consistent with pilot and feasibility trial recommendations, the statistical focus will be descriptive statistics (means, proportions, medians) and differences between the groups and their associated confidence intervals or quantile ranges, rather than repeated *P* value testing [41]. Pilot studies may usefully help assess minimum clinically important differences even if confidence intervals reported are numerically less than 95% (such as 85% or 75%) [37]. Due to this study relying on prescribers correctly transcribing randomization schedules from REDCap into the electronic medical records, our primary analysis will be a per-protocol analysis. We will also conduct an intention to treat analysis for all patients who were eligible and randomized. If a patient is randomized in technical error and was ineligible on the eligibility criteria, they will not be included in the intention to treat analysis. Where a patient is deemed to be in alcohol withdrawal (and managed as such) by the treating physician, and later found not to be an alcohol user, they will be included in the intention to treat analysis. They will be removed in a modified intention to treat (sensitivity) analysis to assess the effect. Given the small sample size and loss of precision of this event, the total sample size may be inflated such that the modified intention to treat analysis includes the original target of 45 patients.

### Progression criteria

The pre-specified thresholds for progression are provided in Table 4 [42, 43].

**Table 4:** Progression criteria for external randomized trial

	**Go—proceed with RCT**	**Amend—proceed with changes**	**Stop—do not proceed unless changes are possible**
**1. Feasibility of patient recruitment** Can 45 patients be recruited in a feasible timeframe?	If 45 patients (100%) are recruited in 24 months from the study start date	If 22–44 patients are recruited in 24 months from the study start date	If fewer than 22 (approximately 50%) patients are recruited in 24 months from the study start date
**2. Feasibility of patient retention** Can 45 patients be retained in the study at its completion?	If 40 patients (approximately 90%) are retained until completion	If 32–39 patients are retained until completion	If less than 32 (approximately 70%) patients retained until completion

There is no dedicated qualitative arm in this trial as given the variety in care delivery models, larger multi-feasibility trials are likely required before efficacy and implementation can be confidently evaluated. The investigators will compile informal conversations with clinical professionals and lessons learned in accordance with best practice.

### Dissemination

Trial results will be disseminated by a final report and sent to participants upon request. Any protocol amendments will be noted in the publication of the results. Further dissemination will include local institute and wider conference presentations, and publication in peer-reviewed journal articles.

## Discussion

Alcohol is the most used and misused drug globally, and alcohol use disorders represent a considerable burden to healthcare systems [1]. Alcohol-use disorders include a spectrum from excessive use, abuse, dependence, to addiction. Patients with alcohol-use disorders are not only at risk of withdrawal symptoms, but these patients have a higher rate of complications, longer duration of ICU and hospital admission, and increased mortality in comparison to patients who do not abuse alcohol [1].

Managing patients with alcohol-use disorders who present to the hospital or ICU can be extremely complicated, as a hospital admission usually results in immediate abstinence from drinking alcohol and can trigger alcohol withdrawal syndrome. While the symptoms vary in intensity, approximately one in five patients can develop seizures, and patients in the ICU who are reliant on life support and who develop delirium due to alcohol withdrawal syndrome may risk harming themselves by accidental removal of catheters or tubes that are providing this life support. An additional complexity with alcohol withdrawal in the critically ill is that alcohol withdrawal is commonly an intercurrent issue and often not the primary diagnosis at admission—and that symptoms of withdrawal (autonomic hyperactivity and delirium) may be masked by another primary diagnosis, comorbidity, or current ICU treatment.

The current mainstay of treatment for alcohol withdrawal is benzodiazepines [44]. This class of drug is known to ameliorate patient agitation, reduce the incidence of alcohol withdrawal seizures, and prevent alcohol withdrawal delirium [45]. However, for many patients, lesser doses of benzodiazepines provide inadequate symptom control, such that larger doses of benzodiazepines are administered. Benzodiazepine drugs in large doses can exacerbate the patient’s acute confusion state (known as delirium), and weaken respiratory effort, leading to mechanical ventilation and prolonged ICU admission [8, 9, 46]. Other pharmacological adjuncts that have been reported to be useful in the management of alcohol withdrawal syndrome include carbamazepine, valproic acid, sodium oxybate, baclofen, gabapentin, and topiramate, but none of these drugs will be tested in this trial [47].

In North America, many centers use phenobarbital for the treatment for alcohol withdrawal, either administered alone or as a benzodiazepine-sparing agent, and it is increasingly being recommended as an alternative agent in guidelines, albeit on quite sparse randomized literature [12, 32, 48–50]. This study will determine whether it is feasible to conduct a trial administering phenobarbital in addition to usual care.

Strengths of our trial include novel data reporting in an ICU population, and the embedding of all aspects of the trial within the electronic medical records with stratified randomization. One-directional communication from the electronic medical record to REDCap to perform real-time randomization. The use of the Epic electronic medical record and alerts to support real-time screening and randomization minimizes human error and saves time for clinical staff, allowing resource allocation of staff to more complex tasks. Limitations of our trial include the open-label design and small sample population being studied. The open-label design increases the risk of expectation bias [51]. However, this was deemed necessary given difficulties with patient recruitment elsewhere (PHENOMANAL trial), complexities of blinding for load plus top-up regimen, and clinical unfamiliarity with phenobarbital in Australia [17]. The sample population being included will inform feasibility, but no inferences will be able to be made about clinical outcomes [52]. A further limitation is the lack of a standardized comparator arm. A comparator group can either be protocolized or usual care (sometimes termed “wild-type” or “care as it is currently provided”) [53, 54]. Protocolized care for the comparator group does improve internal validity, particularly in multicenter trials. Given that there is limited high-quality evidence to support any existing approach to alcohol withdrawal in the ICU this would be favorable. However, this is a single-center trial—where usual care is informed by a policy which the authors deem representative of Australian standard practice. This mitigates some of the risk of heterogeneity of practice.

## Trial status

The recruitment began on 8 July 2026. The trial will be stopped if recruitment is not completed by 7 January 2028.

## Supplementary Information

Supplemental Material 1.

## Data Availability

The datasets used and/or analyzed during the current study are available from the corresponding author on reasonable request.

## References

[1] Mehta AJ. Alcoholism and critical illness: A review. WJCCM. 2016;5(1):27. 26855891 10.5492/wjccm.v5.i1.27 PMC4733453

[2] Australian Institute of H, Welfare. Alcohol, tobacco & other drugs in Australia. Web Report. AIHW; 2024.

[3] Kattimani S, Bharadwaj B. Clinical management of alcohol withdrawal: A systematic review. Ind Psychiatry J. 2013;22(2):100–8. 25013309 10.4103/0972-6748.132914 PMC4085800

[4] Maldonado JR, Sher Y, Das S, Hills-Evans K, Frenklach A, Lolak S, et al. Prospective Validation Study of the Prediction of Alcohol Withdrawal Severity Scale (PAWSS) in Medically Ill Inpatients: A New Scale for the Prediction of Complicated Alcohol Withdrawal Syndrome. Alcohol Alcohol. 2015;50(5):509–18. 25999438 10.1093/alcalc/agv043

[5] Mayo-Smith MF. Pharmacological Management of Alcohol Withdrawal: A Meta-analysis and Evidence-Based Practice Guideline. JAMA. 1997;278(2):144. 9214531 10.1001/jama.278.2.144

[6] Langlois H, Cormier M, Villeneuve E, Hoffman RS, Longo C, Gosselin S. Benzodiazepine resistant alcohol withdrawal: What is the clinician’s preferred definition? CJEM. 2020;22(2):165–9. 31645232 10.1017/cem.2019.421

[7] Löscher W, Rogawski MA. How theories evolved concerning the mechanism of action of barbiturates. Epilepsia. 2012;53(Suppl 8):12–25. 23205959 10.1111/epi.12025

[8] Zaal IJ, Devlin JW, Hazelbag M, Klein Klouwenberg PMC, Van Der Kooi AW, Ong DSY, et al. Benzodiazepine-associated delirium in critically ill adults. Intensive Care Med. 2015;41(12):2130–7. 26404392 10.1007/s00134-015-4063-z

[9] Shafiekhani M, Mirjalili M, Vazin A. Psychotropic drug therapy in patients in the intensive care unit - usage, adverse effects, and drug interactions: a review. Ther Clin Risk Manag. 2018;14:1799–812. 30319262 10.2147/TCRM.S176079 PMC6168070

[10] López-Muñoz F, Ucha-Udabe R, Alamo C. The history of barbiturates a century after their clinical introduction. Neuropsychiatr Dis Treat. 2005;1(4):329–43. 18568113 PMC2424120

[11] Umar Z, Haseeb Ul Rasool M, Muhammad S, Yousaf S, Nassar M, Ilyas U, et al. Phenobarbital and alcohol withdrawal syndrome: a systematic review and meta-analysis. Cureus. 2023;15(1):e33695. 36788902 10.7759/cureus.33695 PMC9922035

[12] Hammond DA, Rowe JM, Wong A, Wiley TL, Lee KC, Kane-Gill SL. Patient Outcomes Associated With Phenobarbital Use With or Without Benzodiazepines for Alcohol Withdrawal Syndrome: A Systematic Review. Hosp Pharm. 2017;52(9):607–16. 29276297 10.1177/0018578717720310 PMC5735736

[13] Malone D, Costin BN, MacElroy D, Al-Hegelan M, Thompson J, Bronshteyn Y. Phenobarbital versus benzodiazepines in alcohol withdrawal syndrome. Neuropsychopharmacol Rep. 2023;43(4):532–41. 37368937 10.1002/npr2.12347 PMC10739082

[14] Law TM, Roper SL, McKinney A, Starnes C, Rains J, Yune JM, et al. Evaluation of Symptom-Triggered Benzodiazepines Versus Phenobarbital for Alcohol Withdrawal Syndrome in Trauma-Surgical Intensive Care Patients. Ann Pharmacother. 2026;60(2):105–14. 40437777 10.1177/10600280251340164

[15] Erfle BA, Steel TL, Law AC, Kaufman DA, Hills-Dunlap K, Afshar M, et al. Pharmacological interventions for alcohol withdrawal syndrome among hospitalized adults: a multicenter cohort study. J Gen Intern Med. 2025. 10.1007/s11606-025-09817-8. 40877712 10.1007/s11606-025-09817-8 PMC12683996

[16] Wolpaw BJ, Oren H, Quinnan-Hostein L, Bradley KA, Johnson NJ, Hallgren KA, et al. Hospital-Wide Implementation, Clinical Outcomes, and Safety of Phenobarbital for Alcohol Withdrawal. JAMA Netw Open. 2025;8(8):e2528694. 40853658 10.1001/jamanetworkopen.2025.28694 PMC12379078

[17] Filewod N, Hwang S, Turner CJ, Rizvi L, Gray S, Klaiman M, et al. Phenobarbital for the management of severe acute alcohol withdrawal (the PHENOMANAL trial): a pilot randomized controlled trial. Pilot Feasibility Stud. 2022;8(1):14. 35065662 10.1186/s40814-021-00963-4 PMC8783453

[18] Macnair A, Love SB, Murray ML, Gilbert DC, Parmar MKB, Denwood T, et al. Accessing routinely collected health data to improve clinical trials: recent experience of access. Trials. 2021;22(1):340. 33971933 10.1186/s13063-021-05295-5 PMC8108438

[19] Mc Cord KA, Ewald H, Ladanie A, Briel M, Speich B, Bucher HC, et al. Current use and costs of electronic health records for clinical trial research: a descriptive study. CMAJ Open. 2019;7(1):E23–32. 30718353 10.9778/cmajo.20180096 PMC6375253

[20] Panganiban HP, Nguyen CD, Abdelhamid YA, Ankravs M, Karahalios E, Macisaac C, et al. Feasibility of Embedding a Randomised Clinical Trial (RCT) into an Electronic Medical Record (EMR) for Patients Admitted to an Intensive Care Unit (ICU). Stud Health Technol Inform. 2024;310:1420–1. 38269676 10.3233/SHTI231224

[21] Appleyard SE, Gilbert DC. Innovative Solutions for Clinical Trial Follow-up: Adding Value from Nationally Held UK Data. Clin Oncol (R Coll Radiol). 2017;29(12):789–95. 29107392 10.1016/j.clon.2017.10.003

[22] Qian ET, Casey JD, Wright A, Wang L, Shotwell MS, Siemann JK, et al. Cefepime vs Piperacillin-Tazobactam in Adults Hospitalized With Acute Infection: The ACORN Randomized Clinical Trial. JAMA. 2023;330(16):1557–67. 37837651 10.1001/jama.2023.20583 PMC10576861

[23] Nguyen CD, Panganiban HP, Fazio T, Karahalios A, Ankravs MJ, MacIsaac CM, et al. A Randomized Noninferiority Trial to Compare Enteral to Parenteral Phosphate Replacement on Biochemistry, Waste, and Environmental Impact and Healthcare Cost in Critically Ill Patients With Mild to Moderate Hypophosphatemia. Crit Care Med. 2024;52(7):1054–64. 38537225 10.1097/CCM.0000000000006255

[24] Council NHaMR. National Statement on Ethical Conduct in Human Research. Council AR, editor. Canberra, Australian Capital Territory: National Health and Medical Research Council; 2023.

[25] Harris PA, Taylor R, Thielke R, Payne J, Gonzalez N, Conde JG. Research electronic data capture (REDCap)–a metadata-driven methodology and workflow process for providing translational research informatics support. J Biomed Inform. 2009;42(2):377–81. 18929686 10.1016/j.jbi.2008.08.010 PMC2700030

[26] Harris PA, Taylor R, Minor BL, Elliott V, Fernandez M, O’Neal L, et al. The REDCap consortium: Building an international community of software platform partners. J Biomed Inform. 2019;95:103208. 31078660 10.1016/j.jbi.2019.103208 PMC7254481

[27] Chan AW, Boutron I, Hopewell S, Moher D, Schulz KF, Collins GS, et al. SPIRIT 2025 statement: Updated guideline for protocols of randomised trials. PLoS Med. 2025;22(4):e1004589. 40294521 10.1371/journal.pmed.1004589 PMC12037212

[28] Wittholz K, Fetterplace K, Karahalios A, Ali Abdelhamid Y, Beach L, Read D, et al. Beta-hydroxy-beta-methylbutyrate supplementation and functional outcomes in multitrauma patients: A pilot randomized controlled trial. JPEN J Parenter Enteral Nutr. 2023;47(8):983–92. 37357015 10.1002/jpen.2527

[29] Brooks L, Reinert JP. Phenobarbital Dosing for the Treatment of Alcohol Withdrawal Syndrome: A Review of the Literature. Journal of Pharmacy Technology. 2024;40(4):186–93. 10.1177/87551225241249407 PMC11325683 39157637

[30] Viswanathan CT, Booker HE, Welling PG. Bioavailability of oral and intramuscular phenobarbital. J Clin Pharmacol. 1978;18(2–3):100–5. 624773 10.1002/j.1552-4604.1978.tb02428.x

[31] Stallworth S, Stilley K, Viriyakitja W, Powers S, Parish A, Erkanli A, et al. Evaluation of phenobarbital dosing strategies for hospitalized patients with alcohol withdrawal syndrome. Gen Hosp Psychiatry. 2023;85:155–62. 37926051 10.1016/j.genhosppsych.2023.10.014 PMC10755809

[32] Hendey GW, Dery RA, Barnes RL, Snowden B, Mentler P. A prospective, randomized, trial of phenobarbital versus benzodiazepines for acute alcohol withdrawal. Am J Emerg Med. 2011;29(4):382–5. 20825805 10.1016/j.ajem.2009.10.010

[33] Shah P, Stegner-Smith KL, Rachid M, Hanif T, Dodd KW. Front-Loaded Versus Low-Intermittent Phenobarbital Dosing for Benzodiazepine-Resistant Severe Alcohol Withdrawal Syndrome. J Med Toxicol. 2022;18(3):198–204. 35668289 10.1007/s13181-022-00900-8 PMC9198182

[34] Nafiu OO, Mills K, Tremper KK. Some Cautionary Tales About Ideal Body Weight Dosing of Anesthetic Medications: It Is Not All That Ideal! Anesth Analg. 2018;127(2):586–8. 29200076 10.1213/ANE.0000000000002662 PMC5976506

[35] Haber PS, Riordan BC, Winter DT, Barrett L, Saunders J, Hides L, et al. New Australian guidelines for the treatment of alcohol problems: an overview of recommendations. Med J Aust. 2021;215(Suppl 7):S3–32. 34601742 10.5694/mja2.51254

[36] Brooke J. SUS: a retrospective. J Usability Studies. 2013;8(2):29–40.

[37] Northcott MH, Johnston G, Presneill JJ, Fazio TN, Adamson N, Ankravs MJ, et al. Aggression, violence and threatening behaviour during critical illness. Crit Care Resusc. 2023;25(2):65–70. 37876598 10.1016/j.ccrj.2023.05.002 PMC10581280

[38] Ankravs MJ, Udy A, Bellomo R, Presneill JJ, Adams L, Abdelhamid YA, et al. Olanzapine versus quetiapine in critically ill patients with hyperactive delirium: Protocol for a multicentre, cluster-randomised, double-crossover, pragmatic clinical trial (CALM-ICU). Crit Care Resusc. 2024;26(4):249–54. 39781498 10.1016/j.ccrj.2024.08.003 PMC11704082

[39] Tyler PD, Du H, Feng M, Bai R, Xu Z, Horowitz GL, et al. Assessment of Intensive Care Unit Laboratory Values That Differ From Reference Ranges and Association With Patient Mortality and Length of Stay. JAMA Netw Open. 2018;1(7):e184521. 30646358 10.1001/jamanetworkopen.2018.4521 PMC6324400

[40] Ying X, Freedland KE, Powell LH, Stuart EA, Ehrhardt S, Mayo-Wilson E. Determining sample size for pilot trials: a tutorial. BMJ. 2025;390:e083405. 40780848 10.1136/bmj-2024-083405

[41] Thabane L, Lancaster G. A guide to the reporting of protocols of pilot and feasibility trials. Pilot Feasibility Stud. 2019;5:37. 30858987 10.1186/s40814-019-0423-8 PMC6393983

[42] Mellor K, Albury C, Dutton SJ, Eldridge S, Hopewell S. Recommendations for progression criteria during external randomised pilot trial design, conduct, analysis and reporting. Pilot Feasibility Stud. 2023;9(1):59. 37061720 10.1186/s40814-023-01291-5 PMC10105402

[43] Mbuagbaw L, Kosa SD, Lawson DO, Stalteri R, Olaiya OR, Alotaibi A, et al. The reporting of progression criteria in protocols of pilot trials designed to assess the feasibility of main trials is insufficient: a meta-epidemiological study. Pilot Feasibility Stud. 2019;5:120. 31700654 10.1186/s40814-019-0500-z PMC6827233

[44] Perry EC. Inpatient management of acute alcohol withdrawal syndrome. CNS Drugs. 2014;28(5):401–10. 24781751 10.1007/s40263-014-0163-5

[45] Mainerova B, Prasko J, Latalova K, Axmann K, Cerna M, Horacek R, et al. Alcohol withdrawal delirium - diagnosis, course and treatment. Biomed Pap Med Fac Univ Palacky Olomouc Czech Repub. 2015;159(1):44–52. 24399242 10.5507/bp.2013.089

[46] Johnson MT, Yamanaka TT, Fraidenburg DR, Kane SP. Benzodiazepine misadventure in acute alcohol withdrawal: the transition from delirium tremens to ICU delirium. J Anesth. 2013;27(1):135–6. 23011118 10.1007/s00540-012-1458-7

[47] Mirijello A, D’Angelo C, Ferrulli A, Vassallo G, Antonelli M, Caputo F, et al. Identification and management of alcohol withdrawal syndrome. Drugs. 2015;75(4):353–65. 25666543 10.1007/s40265-015-0358-1 PMC4978420

[48] Rosenson J, Clements C, Simon B, Vieaux J, Graffman S, Vahidnia F, et al. Phenobarbital for Acute Alcohol Withdrawal: A Prospective Randomized Double-blind Placebo-controlled Study. J Emerg Med. 2013;44(3):592-8.e2. 22999778 10.1016/j.jemermed.2012.07.056

[49] Young GP, Rores C, Murphy C, Dailey RH. Intravenous phenobarbital for alcohol withdrawal and convulsions. Ann Emerg Med. 1987;16(8):847–50. 3619162 10.1016/s0196-0644(87)80520-6

[50] Punia K, Scott W, Manuja K, Campbell K, Balodis IM, MacKillop J. SAEM GRACE: Phenobarbital for alcohol withdrawal management in the emergency department: A systematic review of direct evidence. Acad Emerg Med. 2024;31(5):481–92. 37589203 10.1111/acem.14788

[51] Leon AC, Davis LL, Kraemer HC. The role and interpretation of pilot studies in clinical research. J Psychiatr Res. 2011;45(5):626–9. 21035130 10.1016/j.jpsychires.2010.10.008 PMC3081994

[52] Montgomery R. Sample size justification in feasibility studies: moving beyond published guidance. Pilot Feasibility Stud. 2025;11(1):88. 40551266 10.1186/s40814-025-01675-9 PMC12186404

[53] Turner KM, Huntley A, Yardley T, Dawson S, Dawson S. Defining usual care comparators when designing pragmatic trials of complex health interventions: a methodology review. Trials. 2024;25(1):117. 38342896 10.1186/s13063-024-07956-7 PMC10860249

[54] Ford VJ, Klein HG, Danner RL, Applefeld WN, Wang J, Cortes-Puch I, et al. Controls, comparator arms, and designs for critical care comparative effectiveness research: It’s complicated. Clin Trials. 2024;21(1):124–35. 37615179 10.1177/17407745231195094 PMC10891304

