# Supplementary material for: Phenobarbital as an Adjuvant to benzodiazepines when compared to Single-agent benzodiazepine Treatment (usual care) for Alcohol withdrawal syndrome in the intensive care unit (PASTA): a study protocol
Source: Pilot Feasibility Stud. 2026 Jun 11;12:119. doi: 10.1186/s40814-026-01867-x (PMC13483696; doi:10.1186/s40814-026-01867-x)
Supplement: Supplementary file 1 [file 40814_2026_1867_MOESM1_ESM.docx]

Supplemental Material for Phenobarbital as an Adjuvant to benzodiazepine administration when compared to Single-agent benzodiazepine Treatment (usual care) for Alcohol Withdrawal Syndrome in the Intensive Care Unit (PASTA): a study protocol for a feasibility trial that embeds screening, enrolment and randomization within the electronic medical records

Table of Contents

[Electronic Medical Records (EMR) Embedded Randomized Controlled Trial (RCT)	2](#_Toc206148968)

[Design Process	2](#_Toc206148969)

[Screening	3](#_Toc206148970)

[Figure S1 – EMR silent alert for screening	3](#_Toc206148971)

[Enrolment	3](#_Toc206148972)

[Figure S2 – EMR patient association with the study record	4](#_Toc206148973)

[Randomization	4](#_Toc206148974)

[Figure S3 – Physician alert for patient eligibility review and randomization	5](#_Toc206148975)

[Figure S4 – REDCap randomization form	6](#_Toc206148976)

[Figure S5 – REDCap end of survey completion and physician instruction	7](#_Toc206148977)

[REDCap Project Configuration	8](#_Toc206148978)

[Figure S6 – Physician alert when entering drug being investigated	9](#_Toc206148979)

[Figure S7 – EMR study *orderset* with study medications	10](#_Toc206148980)

[Protocol adherence	10](#_Toc206148981)

[Figure S8 – Physician alert when entering a drug that is not consistent with the allocated arm	11](#_Toc206148982)

[Figure S9 – Physician alert when enrolled participants has potential adverse event/s	12](#_Toc206148983)

[Figure S10 – Study team *inbasket* message for participant monitoring	13](#_Toc206148984)

[Data Collection	13](#_Toc206148985)

[Figure S11 – REDCap case report form (under development)	14](#_Toc206148986)

# **Electronic Medical Records (EMR) Embedded Randomized Controlled Trial (RCT)**

We embedded screening, randomization, recruitment, enrolment, protocol adherence and data collection within our electronic medical records.

# **Design Process**

The Epic electronic medical records system's support guide was followed in developing the concept of the build (Epic Systems Corporation, Wisconsin, USA). Multiple build records were used in the design, such as *OurPractice Advisories*, *OrderSets*, *InBasket*, *Smart Data Element, Extensions* and *Reporting Workbench*. A certified and experienced clinical informatics analyst developed the proof-of-concept. The proof-of-concept design validation and co-design sessions were scheduled with multiple stakeholders. The initial proof-of-concept was demonstrated in a meeting with the Chief Medical Informatics Officer and Head of ICU Research. Feedback from the initial demonstration was incorporated into the proof-of-concept design validation. A total of 6 proof-of-concept validation sessions were conducted: four of these with a Research Fellow who is leading the implementation of the trial to validate the overall design, and two sessions with a Senior Research ICU Pharmacist to validate the study drug build. Three further proof-of-concept co-design sessions were conducted: one session with the Medical Informatician and two sessions with ICU Registrars. The feedback from the co-design session was collected using REDCap surveys. Feedback was consolidated and included in the final proof-of-concept design.

The final design was tested through cycle iterations, utilizing standard testing scripts and electronic medical records testing environments. The change management procedures followed to obtain governance approval for these changes. The team sought approval from the hospital electronic medical records team and presented the change request during the weekly precinct change control meeting. The training plan included a series of training sessions, comprising both face-to-face and online sessions, with the medical and nursing staff. The training materials were in the form of presentations, videos, and printed bedside information. The “post-go-live plan” is to monitor issues with the implementation, followed by operational maintenance and monitoring. A ticket management system will be used to monitor issues and requests for this study and will be conducted by the clinical informatics analyst.

# **Screening**

The electronic medical records silently (not displaying to the physician) screen all patients admitted to the ICU against all inclusion criteria when the physician opens the patient's chart. (Figure S1).

© 2025 Epic Systems Corporation

## **Figure S1 – EMR silent alert for screening**

# **Enrolment**

After screening, the electronic medical records then silently (not displayed to physicians) associate the patient with the study records (Figure S2). This will associate the patient with the electronic medical records study record, which assists in monitoring and reporting.

© 2025 Epic Systems Corporation

## **Figure S2 – EMR patient association with the study record**

# **Randomization**

The electronic medical records then alerts the physician to review exclusion criteria when opening the ‘Orders’ activity (Figure S3). The physician then confirmed the absence of all exclusion criteria to proceed and randomize the patient.

In a scenario in which the physician didn’t click the link to randomize and click any of the options for “Arm 1, Arm 2 or Arm 3”, the participant will be randomized in the electronic medical records but not in REDCap. In another opposite scenario, in which the physician clicks the link to REDCap randomization, but did not transcribe the arm allocation in the electronic medical records alert, the participant will be randomized in REDCap but not in the electronic medical records. These two potential scenarios will be recorded and reported as user randomization errors and monitored by the study team both in the electronic medical records and REDCap. Participants who were not correctly randomized will be recorded as in the intention-to-treat and feasibility analysis, as stipulated in the protocol.

To encourage compliance among trainee physicians, we included a photo of Professor Adam Deane, Head of ICU Research and Deputy Director, on all electronic medical records alerts (see Figure S3).

© 2025 Epic Systems Corporation

## **Figure S3 – Physician alert for patient eligibility review and randomization**

The physician clicks the embedded REDCap hyperlink within the alert to launch the randomization page (Figure S4). To accomplish the stratified randomization, the strata of legal sex will be used to ensure equal treatment arm allocation amongst the participants and achieve a balance between groups. The patient's external ID and legal sex automatically populate fields in REDCap; the patient's external ID will be marked as read-only to prevent users from editing and will potentially prevent randomization errors.

## **Figure S4 – REDCap randomization form**

After clicking “Randomise”, the REDCap form is submitted, and a treatment arm number shown (Figure S5).

## **Figure S5 – REDCap end of survey completion and physician instruction**

The physician returns to the electronic medical records alert, transcribe the arm allocation from the REDCap screen (arm 1, arm2 or arm 3), and click accept on the alert. The drug with each arm allocation remains concealed from the physician during this stage.

The study team receives an “*In-basket*” message when the alert for randomization is triggered to monitor medical staff compliance. During the usual office hours, the study team monitor the message and reviews patient eligibility and progress. If the medical staff screened the patient in error against the inclusion criteria, a randomization alert will be triggered again for the study team to review. The study team will receive similar alert in Figure S3 and will review the eligibility criteria again and randomize the patient correctly.

# **REDCap Project Configuration**

The REDCap project hosted at The Royal Melbourne Hospital will be set up for randomization. The administrator was consulted on the project design to ensure system compliance. The project was configured as a public survey to provide seamless randomization without requiring the physician to log into REDCap and provide user credentials. The public survey will be restricted to physicians and study team, and will only appear to eligible patients based on rules and logic within the electronic medical records alert. The instruments within the project were designed with minimal physician entry and will be configured to automatically retrieve information from electronic medical records and populate REDCap variable. The actions “*@READONLY*” and “*@HIDDEN*” are applied to the instruments to prevent end-user errors. The real-time randomization feature available in version 15 will be used for the project. The “*Submit*” button in the REDCap survey has been changed to “*Randomise*” to be more intuitive for the physician. The end of the survey message has been modified to include instructions on how to transcribe the allocated arm to the electronic medical records alert.

Once the patient is randomized and enrolled an alert (Figure S6) will appear to the physician when they enter a drug that is included in the drug group being investigated within the electronic medical records ‘*Orders*’ activity. The alert advises the physician to remove the entered drug and use the study *OrderSets* to ensure the study drug is ordered according to the participant's allocated arm.

© 2025 Epic Systems Corporation

## **Figure S6 – Physician alert when entering drug being investigated**

Once the physician selects "*Accept*" on the alert, they are directed to a study-specific "*OrderSets*". *OrderSets* is a feature within Epic that can be configured to have a default treatment. For this study, we configured the default treatment according to the participant treatment allocation.

Only at this stage does the physician become aware of the treatment allocation and is instructed to sign for the allocated drug (Figure S7). Due to a lack of funding, it was not possible to administer a blind intervention (i.e., placebo), and it remains a legal requirement that the physician was aware of the drug and dosing before authorizing (signing) the prescription.

© 2025 Epic Systems Corporation

## **Figure S7 – EMR study *orderset* with study medications**

# **Protocol adherence**

If enrolled participants required an additional dose of benzodiazepine and a physician entered a drug within the ‘*Orders*’ activity, they received an electronic medical records alert advising them that the patient was enrolled in this trial and encouraging compliance with the protocol (Figure S8). The physician is advised to remove the entered drug order and open the study *OrderSets*, which contain the allocated treatment.

 

© 2025 Epic Systems Corporation

## **Figure S8 – Physician alert when entering a drug that is not consistent with the allocated arm**

Additional electronic medical records alerts were created to notify physicians when a patient had documented potential adverse events. The physicians were asked to contact the study team to report the event (Figure 9).

 

© 2025 Epic Systems Corporation

## **Figure S9 – Physician alert when enrolled participants has potential adverse event/s**

The study team also receives real-time messages from the electronic medical records alert to monitor the participant's progress within the trial using an electronic medical records “*InBasket*” messaging functionality (Figure S10). Each stage of the participants' progress sends a message to the study team that will be used for monitoring and tracking. The “*Patient screened*” message type will be used to track the number of patients who have been screened. The message type “*Patient considered for randomisation*” will be used to identify patients who have been successfully randomized. The “*Patient enrolled*” message type will be used to identify enrolled participants and to monitor whether the study order has been successfully placed. This message will also be used to monitor discontinued study orders that will be recorded. The message type “*Potential cross-over*” will be used for enrolled participants for monitoring cross-treatment contamination. “*Potential withdrawal due to adverse events*” message type will be used to track enrolled participants who had adverse event occurrences during the intervention period. All the message types contain the name of the physician who triggered the alert, and the action performed on the alert, assisting the study team in monitoring follow-up.

© 2025 Epic Systems Corporation

## **Figure S10 – Study team *inbasket* message for participant monitoring**

# **Data Collection**

For non-binary data variables, automated data extraction will be performed using the “*Reporting Workbench*” and “*Structured Query Language*” tools. Other data variables that require narrative interpretation by the study team will be manually captured and entered into the REDCap study case report forms (under development) (Figure S11).

## **Figure S11 – REDCap case report form (under development)**
